# Predictive intelligence for future vibriosis risk in the eastern United States employing Bayesian spatial modeling

**DOI:** 10.1128/aem.02135-25

**Published:** 2026-03-11

**Authors:** Bailey M. Magers, Sunil Kumar, Kyle D. Brumfield, Katherine Deliz Quiñones, Rita R. Colwell, Antarpreet S. Jutla

**Affiliations:** 1 Geohealth and Hydrology Laboratory, Department of Environmental Engineering Sciences, University of Florida 684895, Gainesville, Florida, USA; 2 Maryland Pathogen Research Institute, University of Maryland 1068 , College Park, Maryland, USA; 3 University of Maryland Institute for Advanced Computer Studies, University of Maryland 271755 , College Park, Maryland, USA; 4 Cell Biology and Molecular Genetics, University of Maryland 1068 , College Park, Maryland, USA; 5 Department of Environmental Engineering Sciences, University of Florida 684895, Gainesville, Florida, USA; Indiana University Bloomington, Bloomington, Indiana, USA

**Keywords:** vibriosis, *Vibrio* spp., *Vibrio*

## Abstract

*Vibrio* spp. infections have increased significantly over recent decades. Advances in remote sensing and understanding of environmental parameters driving increased proliferation of pathogenic *Vibrio* spp. have enabled development of predictive risk models. Here, we implement Bayesian spatial modeling (INLA-SPDE) to forecast infection risks for six vibrio species*—V. alginolyticus*, *V. cholerae* non-O1/non-O139, *V. fluvialis*, *V. mimicus*, *V. parahaemolyticus*, and *V. vulnificus*—and their combined burden, i.e., non-cholera vibriosis, along the United States eastern seaboard. Using reports of vibriosis from 1997 to 2019, we modeled infection risk with a 1-month lead time, assessing 11 environmental and 4 socioeconomic predictors, classifying counties as high, medium, and low risk. The total vibriosis model achieved the highest precision (22.6%) and improved precision over time (slope = 0.71% per year). The predicted probability of vibriosis nearly doubled annually between 1997 and 2019 (93.4% increase). Validation employing 2020–2024 vibriosis data for Florida demonstrated further improvement from the calibration period (slope = 4.37%, precision = 47.0%). Prediction during hurricane season is especially promising, as evidenced for hurricanes Helene and Milton (precision = 65.0%). To assess future climate scenario risk, these models integrated data from Bio-ORACLE v3.0 (CMIP6) under six shared socioeconomic pathways. Results show near-universal, ca. 100%, predicted probability of vibriosis by the end of the century during the peak vibriosis season on the east coast under currently likely scenarios. Given this threat and the promise of the models presented here, development of an early warning system for vibriosis in the eastern United States is deemed critical.

**IMPORTANCE:**

This study provides predictive risk models for vibriosis infections along the eastern United States with a 1-month lead time and demonstrates risk of vibriosis under different climate change scenarios. These efforts may lead to the development of future early warning systems, allowing for mitigation of infections and benefit to public health.

## INTRODUCTION

Species of the genus *Vibrio* comprise ecologically significant bacteria, contributing to carbon and nitrogen cycling (1, 2). Recognized for their mutualistic association with aquatic multicellular hosts (3–10), they include human pathogens, notably *Vibrio cholerae* O1, causative agent of pandemic cholera. Access to water, sanitation, and hygiene infrastructure in developed countries of the world has essentially eliminated risk of cholera. However, non-cholera *Vibrio* infections (vibriosis) persist globally. Several non-cholera *Vibrio* spp. cause acute gastroenteritis, septicemia, and other extra-intestinal infections, primarily associated with consumption of raw and undercooked seafood or contact of an open wound with contaminated water (11). The United States Centers for Disease Control and Prevention (CDC) estimates that 80,000 cases of vibriosis occur in the United States each year, ca. 52,000 of which are caused by consumption of contaminated seafood (11). Vibriosis is a serious infection, with *V. vulnificus* presenting as primary septicemia in >50% of cases and proving fatal in ca. 20% of reported wound infections (11).

Between 1996 and 2010, vibriosis cases in the United States were caused by *V. parahaemolyticus* (ca. 45%), *V. vulnificus* (ca. 19%), *V. alginolyticus* (ca. 11%), and *V. cholerae* non-O1/non-O139 (ca. 9%) (12). However, infections caused by *V. fluvialis* and *V. mimicus* have increasingly been reported in recent years (13). Vibriosis incidence has been documented globally, including Japan and southeast Asia (14), northern Europe (15), Peru and Alaska (16), and Malaysia (17).

Changes in *Vibrio* spp. infection, distribution, and frequency have been linked to environmental factors, notably temperature and salinity (18–20), which make these regions susceptible to proliferation of *Vibrio* spp. (21). Notably, *Vibrio* spp. are autochthonous to estuarine and coastal environments, as well as freshwater and riverine ecosystems globally, thriving in warm, moderately saline water (22). As climate change alters global aquatic ecosystems—characterized by increased variability in salinity, ocean warming, acidification, and deoxygenation—the likelihood of environmental conditions aligning globally to favor proliferation of *Vibrio* spp. and other waterborne pathogens also increases (23). Thus, further increase in reported vibriosis infections is expected, and the future risks of these infections require assessment.

In the United States, reported infections of *V. vulnificus* show a significant northern shift in distribution from 1988 to 2018, and climate projections indicate a high probability of *V. vulnificus* infections in all eastern U.S. states by 2100 under medium-to-high emission scenarios (24). Geographical trends for *V. alginolyticus*, *V. cholerae* non-O1/non-O139, *V. fluvialis*, *V. mimicus*, and *V. parahaemolyticus* have also been reported, all of which show a significant increase in case frequency and distribution from 1990 to 2019 (25). Other investigators have also reported an increased incidence of cholera and non-cholera *Vibrio* spp. infections in recent decades in the United States, Bangladesh, and the coastal North Atlantic (26–28).

Given the global ecological significance of *Vibrio* spp. in aquatic ecosystems, namely carbon and nitrogen cycling, and as commensals of zooplankton, eradication of *Vibrio* spp. is not possible (29, 30). Hence, it is essential to understand those environmental parameters associated with their incidence and proliferation in order to provide an early warning for risk of infection, which is essential for both public health and aquaculture. Optimal conditions for *Vibrio* spp. growth vary depending on genetic composition (8, 31–34). However, several environmental parameters have been identified as heavily influencing their incidence and proliferation (18).

Here, predictive intelligence for vibriosis was developed utilizing known environmental associations, with the goal of providing early warning for infection risk. Similar prior efforts have been made to mitigate the risk of pathogenic *Vibrio* spp., but none have been utilized for real-time prediction of vibriosis risk on large spatial scales. A hypothesis of environmental factors influencing risk of cholera was proposed by Colwell in 1996 and explored in several subsequent investigations (20, 28–30, 35, 36) and employed to mitigate risk of cholera outbreaks in several regions globally (37–41). A few predictive models for non-cholera *Vibrio* spp. have been proposed, e.g., *V. parahaemolyticus* (42, 43) and *V. vulnificus* (44). However, they were calibrated for single regions, and capacity for large-scale prediction is needed. Following the Colwell (29) proposal to employ remote sensing data for *Vibrio* spp. investigations, earth observation satellite products have been used to develop predictive models for *V. parahaemolyticus* in Chesapeake Bay (45), non-cholera *Vibrio* spp. in the Baltic Sea (46), and *V. vulnificus* in counties of interest in Hawaii, Alabama, Florida, Louisiana, Mississippi, Texas, Maryland, and New York (47).

Recent research highlights the potential of combining environmental monitoring, particularly via Earth observation technology, with machine learning algorithms to model vibriosis infections in coastal areas (25). Thus, we utilized a variety of environmental and socioeconomic data sets and Bayesian spatial modeling to develop risk prediction models for vibriosis infections in the eastern United States. The models are capable of forecasting risk of infection from *V. alginolyticus*, *V. cholerae* non-O1/non-O139, *V. fluvialis*, *V. mimicus*, *V. parahaemolyticus*, and *V. vulnificus*. We also utilize the models to predict vibriosis infection risk by mid- and end-of-century, based on projections of global aquatic environment alteration, and determine risk of emission scenarios, supporting continued action to limit worst-case scenarios of vibriosis.

## MATERIALS AND METHODS

### Vibriosis case data

Data for reported vibriosis (non-cholera) in the United States were provided by the Centers for Disease Control (CDC) Cholera and Other Vibrio Illness Surveillance (COVIS) system (48). The included the number of reported cases between 1990 and 2019, along with the date when the patient arrived at the hospital, whether the case was confirmed or probable, causative agent, location of the hospital where the case was reported, travel history, and transmission route (foodborne, likely foodborne, nonfoodborne, likely nonfoodborne, or unknown source). Both foodborne and nonfoodborne cases (i.e., total cases) were used for modeling, as previous models using only nonfoodborne infections did not show improvement over total cases (25).

**Fig 1:**
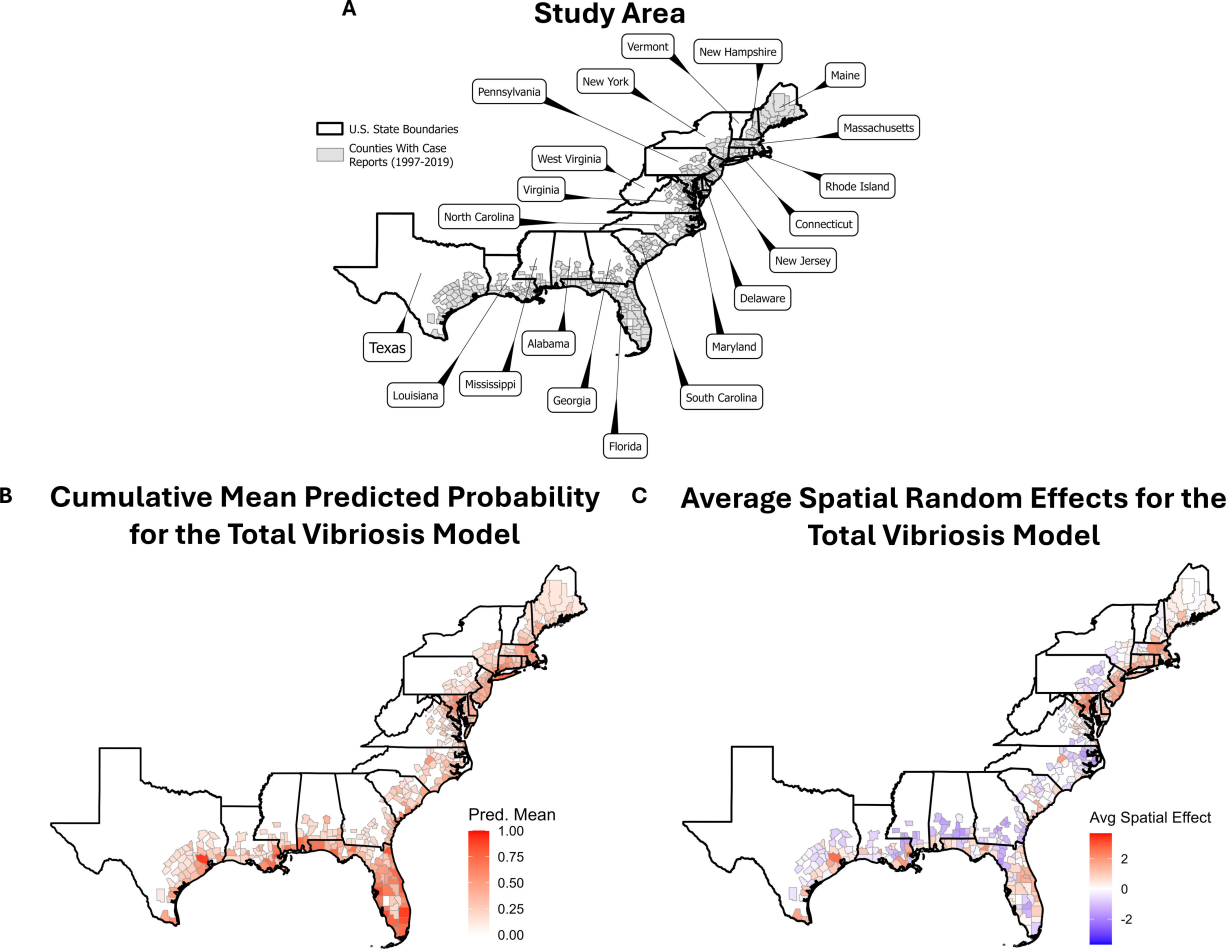
(**A**) Depiction of study area, including all U.S. counties within 200 km of the Atlantic and Gulf coastlines that have reported vibriosis cases (1997–2019), with the names of the respective states displayed. (**B**) Cumulative mean predicted probability of vibriosis for the total vibriosis model, with predicted probability from 0 to 1 (0% to 100% probability of presence). Includes testing data predictions for all available counties (*n* = 405 counties). (**C**) Cumulative mean spatial random effects for the total vibriosis model. Spatial effects represent residual spatial autocorrelation not explained by measured environmental variables. Values indicate areas with higher or lower vibriosis risk than expected based on fixed predictors. The maps were created using the R package tigris.

For this study, both confirmed and probable cases of vibriosis caused by *V. alginolyticus*, *V. cholerae* non-O1/non-O139, *V. fluvialis*, *V. mimicus*, *V. parahaemolyticus*, and *V. vulnificus* were considered. However, it should be noted that of the ca. 10,000 total cases, only ca. 1,000 were categorized as probable cases. Cases in which the patient had traveled recently were omitted from analysis, as were cases with unknown patient travel status. Locations of each case were given on varying spatial scales, with some cases reported at the county level, some at the city level, and others at the state level. Hence, state-level cases were not considered because of coarse spatial resolution. City-level cases were converted to county level, using the county of the identified city for each case. Each case was assigned latitude and longitude location, based on the centroid of each U.S. county where the case was reported. Only cases with assigned latitude and longitude within 200 km of the Atlantic and Gulf Coast of the United States were considered, in accordance with previous studies (24, 25). Figure 1A depicts the study area, including the 405 U.S. counties reporting vibriosis cases along the eastern seaboard.

National monthly vibriosis case data were unavailable post-2019 for model validation. However, monthly data for vibriosis infections were available from some state health departments. To validate the models developed in this study, vibriosis infections (excluding cholera) and *V. vulnificus* infections were used. Data for individual *Vibrio* spp., except *V. vulnificus*, were unavailable; therefore, only *V. vulnificus* and general vibriosis models were validated. Monthly data for each Florida county were available from 2020 to 2024 (49). While total case counts were reported, detailed surveillance data (e.g., mode of infection) were unavailable.

### Environmental data sets

Several environmental parameters were identified as potential predictors to model vibriosis. Sea surface temperature (SST) was found to be directly correlated with outbreaks of cholera (20), and warmer water temperatures have been correlated with increased numbers of non-cholera *Vibrio* spp. (50). Similarly, ranges of sea surface salinity (SSS) have been associated with the abundance of *Vibrio* spp., and concerns have been raised with respect to rising sea surface levels and changes in precipitation patterns that impact salinity and, consequently, the incidence of pathogenic *Vibrio* spp. (20, 27, 50, 51).

Chlorophyll-a (chl-a) has been shown to have an association with both cholera (20, 52–54) and non-cholera *Vibrio* spp. (55–57). Chl-a concentrations have been used to estimate nutrient load and phytoplankton blooms, with the latter serving as a proxy for incidence of zooplankton (20, 29, 58). Since zooplankton, notably copepods, provide a nutrient-rich niche with which *Vibrio* spp. are associated (59, 60), chl-a can also serve as a useful indirect indicator of *Vibrio* incidence when appropriate lag times are considered (61). However, given the typical nonlinear relationship between chl-a and *Vibrio* spp., we derived measurements of phytoplankton divided by size classification from chl-a to explore the potential for more linear indicators, as in our previous work (25).

More directly, nutrients, namely nitrate (NO_3_⁻) and phosphate (PO_4_^−3^), have been shown to have negative and positive associations with *Vibrio* spp., respectively (62–65). Increased dissolved organic matter is associated with enhanced growth of *V. parahaemolyticus* (58). Nitrate, phosphate, and organic matter are required for necessary biological functions of *Vibrio* spp. While some parameters may be utilized as a general proxy for these nutrient concentrations, direct nutrient input from stormwater, agricultural, or urban runoff can be independent of other factors. Thus, direct nutrient inclusion may inform models beyond standard parameters.

Dissolved oxygen (DO) is another indicator of Vibrio abundance, having been negatively correlated with incidence of *Vibrio* spp. in a number of aquatic ecosystems globally (66). In addition, extreme precipitation events have been linked to both cholera (38, 67, 68) and non-cholera *Vibrio* spp. infections (69). Anomalous precipitation was found to alter the salinity of coastal water (70), and the intensity and frequency of extreme events are expected to increase with climate change (71). pH, which is also influenced by precipitation variability, has been shown to have both positive and negative association with the incidence of *Vibrio* spp., depending on the species and geographical area under study (66).

All environmental data were extracted from within 20 km of the U.S. eastern coast (including the Gulf of Mexico), comprising SST, SSS, pH, DO, phytoplankton concentration (separated by size classification), NO_3_⁻, PO_4_^−3^, colored dissolved organic matter (CDOM), and precipitation. These data were converted to monthly averages prior to subsequent analysis. SST data from 1997 to 2024 were compiled from two different sources. Data from 1997 to July 2002 were obtained from the NOAA Optimum Interpolation Sea Surface Temperature (OISST) data set, using the Advanced Very High-Resolution Radiometer (AVHRR) and Visible Infrared Imaging Radiometer Suite (VIIRS) (72). Data from August 2002 to 2024 were sourced from the Aqua-Modis daytime 4 km monthly SST data set, using the NASA Ocean Color Level 3 and 4 data browser (73). Although the Aqua-MODIS data set has a much higher spatial resolution than the OISST data set, the Aqua-MODIS data records began in 2002; hence, two different data sets were used to ensure adequate temporal coverage. Data for SSS were sourced from the Ocean Reanalysis System 5 (ORAS5) global ocean reanalysis monthly data set (74). Precipitation data were obtained from the ERA5 monthly averaged total precipitation data sets (75).

Data for NO_3_⁻, PO_4_^−3^, DO, and pH from 1997 to 2022 were gathered from the Global Ocean Biogeochemistry Analysis and Forecast (76) and the Global Ocean Biogeochemistry Analysis and Forecast for 2023–2024 (77). CDOM and chl-a concentrations, used to calculate phytoplankton size classification (micro-, nano-, and picophytoplankton) using transformations from Hirata et al. (78), were obtained from the Copernicus GlobColour Level 4 monthly interpolated biogeochemical data set provided by the Copernicus Marine Data Store (79). These data were from September 1997 to 2024. Other remote sensing data sources for chl-a concentrations were not readily available prior to this date. The level 4 data set integrates chl-a observations from multiple satellite sensors, including SeaWiFS, MODIS, MERIS, VIIRS (SNPP and JPSS1), and OLCI (S3A and S3B), to produce a continuous, blended product improving spatial and temporal accuracy.

While alternative sensors and data sets exist for these environmental parameters, this study prioritized data sets offering continuity and consistent spatial resolution to enhance accuracy and minimize noise introduced by varying algorithms. The exception was SST, for which the data prior to 2002 were obtained from AVHRR-VIIRS at coarser 0.25° resolution, whereas post-2002 data were obtained from finer 4 km resolution sensors like MODIS. Although previous studies indicated that temperature resolution may have little impact on identifying environmental associations and model performance (20, 40), higher-resolution data were employed.

### Socioeconomic data sets

Four socioeconomic variables were selected as potential model parameters. To approximate the risk of foodborne infections, data from the US Census Bureau County Business Patterns were utilized, including data from 1997 to 2023 (80). Standard Industrial Classification (SIC) codes (1997–1998) and North American Industry Classification System codes (1999–2023) were utilized to determine the total number of fish and seafood markets (SIC codes 5146 and 5421, NAICS codes 445220 and 424460) and seafood product preparation and packaging facilities (SIC codes 2091 and 2091, NAICS codes 311710, 311711, and 311712) for each county. The county totals per year were designated as the total number of seafood establishments in the model.

To approximate the risk of nonfoodborne infections, the number of swim season days for each county was gathered from the U.S. Environmental Protection Agency (EPA) Beach Advisory and Closing On-line Notification (BEACON) 2.0 beach profile list (81). Data for 2000–2025 are included in the beach profile list. The number of swim season days used in the model was the average swim season days across all beaches in each county each year.

To estimate additional socioeconomic vulnerabilities to vibriosis in each county, the CDC social vulnerability index (SVI) was used, specifically the R_PL_THEMES overall percentile ranking for each county (82). SVI data were available for 2000, 2010, 2014, 2016, 2018, 2020, and 2022. SVI was included to identify the impact of infection risk on disadvantaged populations that would not be accounted for by other indicators.

Finally, to estimate the impact of population clusters on reported vibriosis cases, population density was calculated using the 2000, 2010, and 2020 U.S. census (83). Any years or counties that lacked data for socioeconomic parameters from 1997 to 2024 had those years’ data estimated using linear interpolation from the trends of available data, including hindcast or forecast of periods prior to or after available data. If data were not available for a county, such as a lack of swim season days for non-coastal counties, the value was assumed to be zero for the county for the entirety of the study period.

### Bayesian modeling framework

To model vibriosis infections in the eastern United States, a Bayesian spatial modeling approach was utilized. The approach adopted here followed Moraga et al. (84), who modeled malaria using integrated nested Laplace approximation (INLA) in combination with stochastic partial differential equation (SPDE). Model implementation employed the R package R-INLA. Here, we utilized the same methodology in a novel approach for vibriosis modeling. We used the same model and code for R-INLA as Moraga et al. (84), except for the max edge of the spatial mesh, which was set to c(1, 15). This coarser mesh was used to limit computational requirements, given the large spatial extent of the study area.

The calibration modeling period was October 1997 to December 2019, beginning in October 1997, based on environmental data availability. Monthly data were provided to the model for all counties reporting vibriosis cases. The dependent variable was binary, indicating vibriosis presence (1) and absence (0), with presence assigned to county-months where one or more cases were reported. Independent variables included all environmental (lagged by 1 month prior) and socioeconomic parameters (given as annual values with no lag). All independent variables were scaled (given a mean of 0 and standard deviation of 1) so that the model coefficient outputs could be interpreted relative to each other (i.e., determining strength of each variable contribution to model prediction). To account for seasonality not captured by environmental variables, two non-scaled temporal independent variables were included, which were sin (SinMonth) and cos (CosMonth). These variables identify data point location in the annual cycle. Finally, fixed effects from the intercept (b0) and random effects from the spatial Gaussian random field (s) were included.

Several model iterations were created to develop an ensemble model. The data set was split so that 70% of the data were used to train the models and 30% to test model performance on unseen data. This split was performed 10 times for sufficient random sampling. Vibriosis presence was relatively rare (ca. 4% presence of vibriosis in all county-months during the study period, and <1% for *V. alginolyticus*, *V. cholerae*, *V. fluvialis*, and *V. mimicus*), creating a class imbalance of vibriosis presence and absence data for model calibration. Hence, random undersampling of the majority (absence) class was utilized for model training. For each of the 70/30 training/testing splits, 10 random undersamplings of the training data set were performed; thus, 100 iterations of the model were created. Random undersampling was not performed on the testing data set so that each model could be tested on real-world imbalanced data sets, providing accurate measure of true model performance.

### Parameter selection

To determine which independent variables would be included in the model, each variable was added individually to the model formula for the presence/absence of vibriosis caused by each of the six *Vibrio* spp. (general vibriosis model). The base model included the intercept (b0), spatial random effects (s), and seasonality terms. Addition of other variables required improving model precision (measured at a probability threshold of 0.5). This requirement was selected to meet the goal of providing predictions that will optimally allocate and utilize resources. Different orders of variable addition were utilized, with the highest precision improvements determining variable addition order. Three 70/30 splits and three random undersampling iterations (nine total iterations) were used for parameter selection. The average precision across all iterations was used for selection criteria. Environmental parameter selection was performed first, followed by socioeconomic parameter selection using the optimal combination of environmental parameters. Each model, including the individual *V. alginolyticus*, *V. cholerae* non-O1/non-O139, *V. fluvialis*, *V. mimicus*, *V. parahaemolyticus*, and *V. vulnificus* models, as well as the total vibriosis model, had its own parameter selection analysis.

### Relative risk model

Rather than defining a single predicted probability threshold from the model results to inform prediction of vibriosis, we adopted a risk model approach, utilizing relative risk ratios and categories of low, medium, and high risk. This approach was adopted for this study because precision using a binary outcome (presence/absence) will be much lower than risk gradient with multiple categories and increasing certainty. While we selected three risk categories for analysis, these categories serve as general guidelines, with increasing relative risk indicating increased probability of a case being reported. Relative risk ratios were calculated by dividing a predicted probability from a single prediction by the cumulative mean predicted probability of all predictions. Relative risk ratios between models were not comparable, as they are relative to their own cumulative mean predicted probability.

Relative risk thresholds to define low, medium, and high risk for each model were determined as follows. First, relative risk was computed for all predictions to test different thresholds. Threshold testing started at a relative risk of 0 and increased by 0.1 increments until the threshold was above all predictions. Sensitivity at each was used to determine the threshold for low, medium, and high risk. For each model, the threshold for low risk was set at the relative risk value closest to sensitivity of 0.8 (i.e., 20% of reported cases within low risk prediction). Medium risk was set at the value closest to sensitivity of 0.35 (i.e., 35% of reported cases within medium risk prediction and 45% within high risk prediction). High risk was defined as exceeding this relative risk threshold. The sensitivity values were selected because most reported cases should be designated high risk among the three categories, with relatively few classified as low risk. Raising the threshold for high risk would increase model precision but result in more cases being overlooked. Conversely, lowering the threshold ensured that more cases were captured by the model, but precision would be lower. Thus, the thresholds selected captured the balance between accurate prediction and missed prediction. Once thresholds for each model were identified, predictions were categorized into appropriate risk categories for performance analyses, including precision, sensitivity, and prediction-to-case ratio (i.e., number of total predictions divided by number of true positives, or the inverse of precision).

### Model validation

To test model performance post-2019, the vibriosis case data used were provided by the Florida Department of Health (FDOH) for 2020–2024, for both total vibriosis (excluding cholera) and *V. vulnificus*. It should be noted that monthly data from COVIS and FDOH do not match pre-2019 for Florida cases. This discrepancy may reflect differences between the national and state-level surveillance and reporting practices. Details regarding this discrepancy are provided in the supplemental material. While this discrepancy exists, the impact of validation analysis remains, since better local monitoring should provide more realistic assessment of how the model performs. The data for other individual *Vibrio* spp. were not available.

The 100 trained models were used to predict the relative risk of vibriosis in each county for each month, utilizing 1-month lagged environmental data sets from the same sources as the trained models (see “Environmental data sets,” above). Data sources for socioeconomic variables were also unchanged, with socioeconomic data again determined for missing years with linear interpolation and linear forecasting if necessary. Predictions obtained from the models were averaged for each county-month to determine the predicted probability and relative risk for each prediction. Relative risk ratios were measured using the same cumulative mean predicted probability as in training/testing to ensure consistency. Thus, prediction is relative to historic risk for each county. Each prediction was assigned a risk category based on previously identified thresholds. Precision, sensitivity, and prediction-to-case ratios were computed for both models at low, medium, and high risk.

### Prediction of future vibriosis risk

To assess future risk of vibriosis under different future climate scenarios, environmental data were gathered from Bio-ORACLE v3.0 (85). Bio-ORACLE provides data for all marine environmental parameters included in this study (provides chl-a to compute phytoplankton by size classification), utilizing data of the Sixth Phase of the Coupled Model Intercomparison Project (CMIP6) and several shared socioeconomic pathways (SSPs) for its projections. Data projections are reported per decade, starting with 2020–2030 and ending with 2090–2100.

However, the data provided by Bio-ORACLE could not be directly integrated into the models because certain parameters, namely DO and pH, appeared to be outside the range of the trained data used for the models, even the baseline years. These discrepancies caused large over-prediction of risk in baseline years when using Bio-ORACLE. Therefore, to integrate projections of the Bio-ORACLE data effectively, linear trends of Bio-ORACLE environmental data associated with each U.S. county (using the five nearest data points within 20 km of the U.S. eastern coastline, as with previous environmental data used for the models) were computed on a per-county basis, utilizing the mean parameter projection for each available decade (2020–2100) and each SSP scenario, including SSP1-1.9, SSP1-2.6, SSP2-4.5, SSP3-7.0, SSP4-6.0, and SSP5-8.5. Each SSP presents a different scenario of climate warming and societal response. The linear trends for each county were used to project environmental parameter values of the trained data sets employed for model calibration to maintain data consistency.

For projections, July 2050 and July 2100 were selected as the months of interest, July having the highest predicted probability of total vibriosis case reports. Projections were limited to counties having reported vibriosis cases. Values for July 2050 and 2100 were calculated using previously computed linear trends and environmental data from July 2019 (last year of the calibration period) as baseline for prediction.

Socioeconomic data projections for the total vibriosis model, which includes swim season days and total seafood establishments as parameters, are not available. Projections were made using county-level linear trends to forecast each parameter for July 2050 and 2100 using available socioeconomic data. As this methodology carries high uncertainty, sensitivity analysis was conducted, with projected models run using both constant (data from the 2019 baseline) and projected socioeconomic data.

## RESULTS

### Model parameters

Each model had a unique combination of environmental and socioeconomic parameters, and the parameter coefficients and their significance varied. Table 1 displays selected parameters for each model, including mean, standard deviation, and 95% confidence intervals of the parameter coefficients. Detailed information on parameter demographics is included in the supplemental material. All parameters were included in at least one model. The intercept, b0, had a strong negative coefficient in each model, indicating low baseline probability for infection. CosMonth was strongly negative in six of seven models, and SinMonth was strongly negative in four of seven models, indicating a peak in predicted probability in the summer (June–August) and lowest predicted probability in winter (December–February). The *V. cholerae* and *V. mimicus* models had positive SinMonth coefficients, indicating peak and trough shifted earlier in the year by a month or two.

**TABLE 1:** Ensemble fixed effect summaries for each model, including mean, standard deviation (Std), and 95% confidence intervals (CI) for each model fixed effect parameter^*a*^

Parameter	Mean coeff	Std coeff	Lower 95% CI	Upper 95% CI
Total vibriosis				
DO	0.902	0.146	0.504	1.302
SST	0.878	0.089	0.653	1.106
Swim season	0.365	0.039	0.229	0.498
Total seafood establishments	0.352	0.051	0.237	0.467
PO4	−0.067	0.309	−0.960	0.824
Picophytoplankton	−0.194	0.037	−0.294	−0.095
SinMonth	−0.674	0.113	−0.997	−0.345
pH	−0.826	0.063	−0.987	−0.665
NO3	−0.957	0.261	−1.717	−0.196
CosMonth	−1.019	0.065	−1.185	−0.853
b0	−1.289	0.042	−1.543	−1.044
*V. alginolyticus*				
SST	1.011	0.175	0.538	1.499
Total seafood establishments	0.525	0.140	0.309	0.745
Swim season days	0.486	0.075	0.277	0.696
DO	0.455	0.235	−0.263	1.186
Microphytoplankton	−0.113	0.087	−0.340	0.115
CDOM	−0.119	0.078	−0.365	0.129
Social vulnerability index	−0.538	0.090	−0.806	−0.277
NO3	−0.689	0.227	−1.266	−0.126
CosMonth	−0.816	0.135	−1.174	−0.461
SinMonth	−1.273	0.257	−1.945	−0.590
pH	−1.319	0.141	−1.690	−0.955
b0	−1.679	0.095	−2.133	−1.247
*V. cholerae*, non-O1/non-O139				
Swim season days	0.439	0.129	0.142	0.755
SinMonth	0.367	0.242	−0.310	1.050
SST	0.136	0.024	0.065	0.210
Total seafood establishments	0.042	0.019	0.016	0.068
Population density	0.000	0.000	0.000	0.000
CosMonth	−0.796	0.169	−1.212	−0.392
b0	−4.132	0.569	−5.838	−2.542
*V. fluvialis*				
DO	0.935	0.336	0.061	1.824
SST	0.818	0.208	0.247	1.405
Total seafood establishments	0.530	0.169	0.239	0.825
Swim season days	0.199	0.099	−0.072	0.476
Picophytoplankton	−0.175	0.117	−0.449	0.094
Social vulnerability index	−0.370	0.141	−0.703	−0.045
CosMonth	−0.611	0.188	−1.079	−0.151
SinMonth	−0.749	0.261	−1.550	0.043
pH	−0.927	0.169	−1.393	−0.474
b0	−0.941	0.114	−1.375	−0.561
NO3	−1.125	0.275	−1.854	−0.411
*V. mimicus*				
SST	1.687	1.892	0.033	4.226
Total seafood establishments	1.158	1.638	0.082	2.842
SinMonth	0.996	2.571	−1.101	3.351
Swim season days	0.121	0.320	−0.656	0.977
Social vulnerability index	−0.271	0.561	−1.162	0.588
pH	−0.519	0.884	−1.686	0.447
CosMonth	−0.882	1.291	−2.336	0.074
b0	−1.532	0.835	−3.778	−0.199
*V. parahaemolyticus*				
DO	1.075	0.189	0.557	1.601
SST	0.792	0.139	0.472	1.121
Total seafood establishments	0.467	0.099	0.298	0.637
Swim season days	0.394	0.061	0.223	0.565
CDOM	0.016	0.057	−0.134	0.166
Population density	−0.004	0.045	−0.140	0.137
Nanophytoplankton	−0.027	0.053	−0.185	0.131
Precipitation	−0.093	0.047	−0.201	0.014
SSS	−0.103	0.085	−0.417	0.203
Picophytoplankton	−0.204	0.062	−0.363	−0.046
Social vulnerability index	−0.340	0.064	−0.538	−0.146
SinMonth	−0.756	0.204	−1.216	−0.287
pH	−0.885	0.081	−1.131	−0.641
CosMonth	−1.125	0.090	−1.366	−0.886
NO3	−1.287	0.155	−1.717	−0.867
b0	−1.367	0.051	−1.675	−1.079
*V. vulnificus*				
Swim season days	0.537	0.089	0.335	0.738
Microphytoplankton	0.262	0.073	0.054	0.472
SSS	−0.057	0.164	−0.578	0.446
pH	−0.162	0.110	−0.421	0.096
Social vulnerability index	−0.214	0.077	−0.438	0.012
CosMonth	−1.250	0.092	−1.478	−1.024
SinMonth	−1.495	0.149	−1.856	−1.137
b0	−1.630	0.101	−2.583	−0.857

^*a*^ Parameters with significant coefficients (*P* < 0.05) are highlighted.

Overall, two environmental parameters, DO and SST, showed strong positive coefficients, while NO_3_⁻ and pH had strong negative coefficients. Other parameters had a generally weaker effect on model output. Of the environmental variables, SST and pH were included in six of the seven models, while PO_4_^−3^ and precipitation were included in only one of the seven. For socioeconomic variables, swim season days and total seafood establishments generally showed strong positive coefficients, while SVI had a mix of strong and weak negative coefficients and population density had minimal effect. Of socioeconomic variables, swim season days and total seafood establishments were included in most models (all seven and six of seven, respectively), while population density was included in the fewest models (two of seven). The *V. cholerae* model included one environmental and three socioeconomic parameters, while the *V. parahaemolyticus* model included the most, with eight environmental and four socioeconomic parameters.

### Spatial and temporal hotspots

Figure 1B displays cumulative mean predicted probability for the total vibriosis model. Results for the individual *Vibrio* spp. models are provided in the supplemental material. Hotspots of high vibriosis probability were detected along the coast of Florida. The Gulf Coast was found to be a regional hotspot as well. Other notable hotspots included Long Island, New York, eastern Massachusetts, southwest Connecticut, Savannah, Georgia, Charleston, South Carolina, and northern Chesapeake Bay. The hotspots varied across individual species models. For example, the probability of *V. mimicus* and *V. cholerae* non-O1/non-O139 was highest along the Gulf Coast, relative to other models. The probability of *V. fluvialis* and *V. parahaemolyticus* was higher in the northeast but much lower in the northeast for *V. vulnificus*.

Model spatial random effects also informed regional hotspots by accounting for spatial autocorrelation and effects not modeled by fixed model parameters, which can increase or decrease probability of cases in certain regions. Figure 1C shows the average spatial effects for the total vibriosis model. Results for individual species are included in the supplemental material. All models, apart from *V. vulnificus*, indicated strong positive spatial effect in the northeast United States, suggesting that some variables contribute to increased probability of vibriosis in regions not included in the selected variables. Additionally, most models showed strong positive spatial effects for Florida and along the Gulf Coast. Notably, most models indicated negative spatial effect for the Mid-Atlantic region (Georgia, South Carolina, North Carolina), suggesting an unmodeled variable muting the probability of reported cases. Potential unmodeled variables may include local environmental changes or processes not easily observed by coarse satellite monitoring or anthropogenic factors. This includes factors such as recreational activity or coastal development that are not captured by proxies like beach or seafood establishment data. Another potential factor is regional surveillance robustness, which was not accounted for in the model.

**Fig 2:**
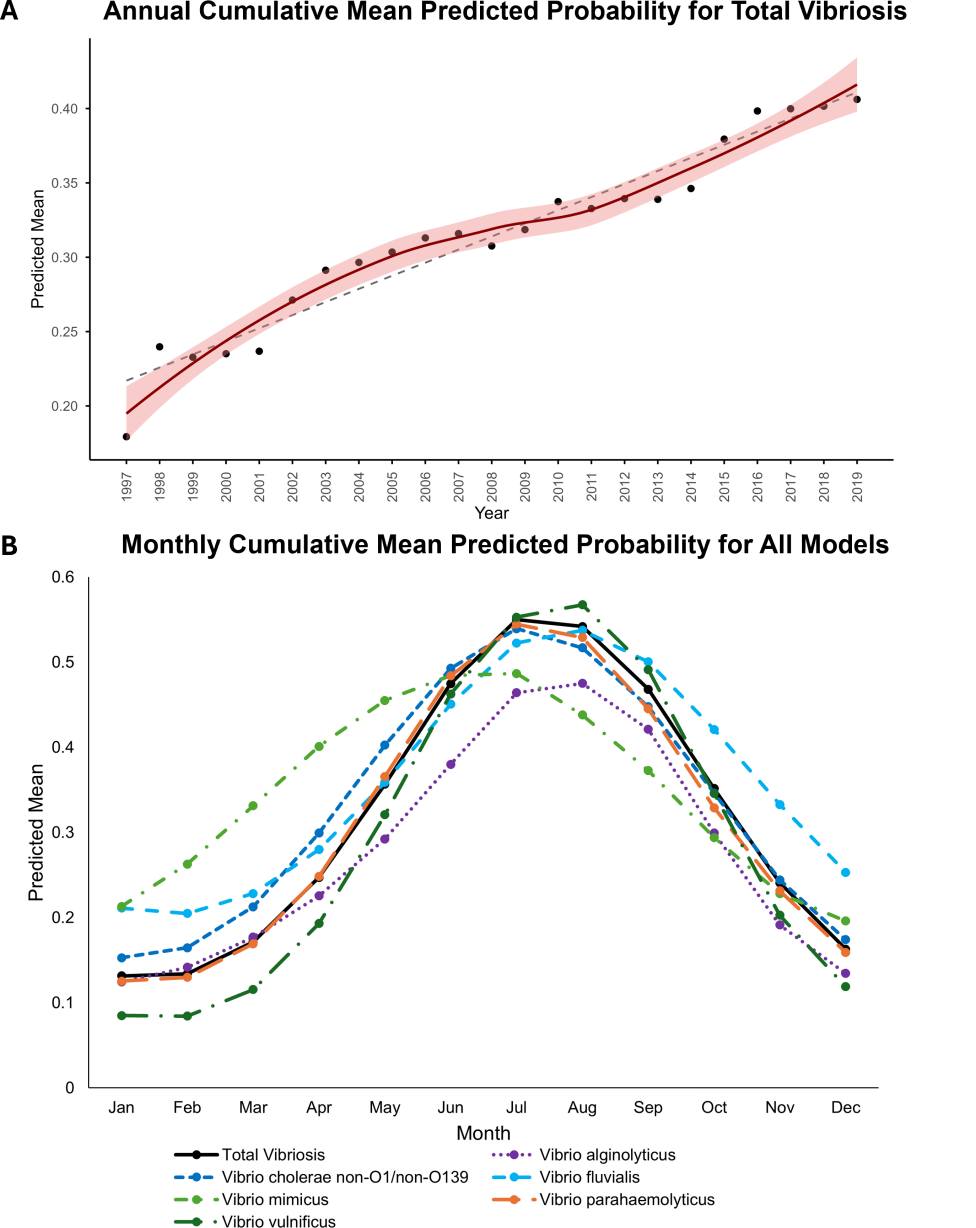
Cumulative mean predicted probability. (**A**) Annually for total vibriosis, including linear trendline (shown as dashed line, computed using linear regression) and nonlinear trend (shown in red with 95% confidence interval [CI]) computed using LOESS (span = 0.8), and (**B**) monthly for all models, with predicted probability from 0 to 1, (0% probability of presence to 100% probability). Includes testing data predictions for all available counties (*n* = 405 counties).

Noticeable trends in predicted probability were both annual and seasonal. Figure 2A shows annual cumulative mean probability for total vibriosis from 1997 to 2019. Predicted probability (also relative risk) increased significantly over time. The trend is primarily linear, with *R*^2^ of 0.94, but there is some nonlinear variation, as depicted by the nonlinear Locally Estimated Scatterplot Smoothing (LOESS) in Fig. 2A. From the linear trendline equation, predicted probability increased by ca. 0.009 (ca. 0.9%) annually. Given the 23-year study period of 23 years and intercept of 0.217, the probability of vibriosis caused by any of the species approximately doubled post-1997, on average, across the entire eastern United States. Figure 2B shows seasonal trends in predicted probability, with peaks for most models in July or August and troughs from December to February.

### Risk model performance evaluation

Table 2 displays risk thresholds, precision, sensitivity, and prediction-to-case ratio for each risk category of all models. Sensitivity for each risk category is in accord with sensitivity used to determine threshold (ca. 20% for low, ca. 35% for medium, and ca. 45% for high). The total vibriosis model indicated the highest precision of high risk (22.61%), followed by *V. parahaemolyticus* (13.73%), *V. alginolyticus* (10.05%), *V. vulnificus* (9.38%), *V. cholerae* (2.70%), *V. fluvialis* (2.53%), and *V. mimicus* (1.79%). The total vibriosis model was best performing, according to this metric, with approximately one of every four counties predicted to be at high risk for vibriosis case reports. With respect to areas of low risk, the total vibriosis model achieved precision of 99.17%, with only one of every 120 counties predicted to be at low risk that month. Also notable was improvement for high-, medium-, and low-risk categories, with high-risk predictions 3.2–8.5 times more precise than medium-risk predictions and 25.3–47.9 times more precise than low-risk predictions, depending on the model.

**TABLE 2:** Precision, sensitivity, and prediction-to-case ratio (i.e., number of total predictions divided by reported cases within the risk category and rounded to nearest whole number) for all models by risk category, including performance for both COVIS and FDOH data

Species/model	Risk category	Relative risk range	Precision	Sensitivity	Prediction-to-case ratio
1997–2019 (COVIS)					
Total vibriosis	High	≥2.5	22.61%	44.00%	4
	Medium	1.6–2.5	6.91%	33.97%	14
	Low	<1.6	0.83%	18.96%	120
*V. alginolyticus*	High	≥3.0	10.05%	45.34%	10
	Medium	1.9–3.0	2.28%	32.69%	44
	Low	<1.9	0.21%	18.52%	479
*V. cholerae*, non-O1/non-O139	High	≥2.2	2.70%	43.88%	37
	Medium	1.3–2.2	0.49%	34.31%	203
	Low	<1.3	0.10%	19.15%	1,029
*V. fluvialis*	High	≥2.0	2.53%	42.43%	40
	Medium	1.2-2.0	0.59%	38.99%	169
	Low	<1.2	0.10%	16.51%	976
*V. mimicus*	High	≥2.2	1.79%	42.59%	56
	Medium	1.3–2.2	0.21%	35.19%	473
	Low	<1.3	0.04%	20.37%	2,289
*V. parahaemolyticus*	High	≥2.5	13.73%	44.42%	7
	Medium	1.7–2.5	4.26%	32.31%	23
	Low	<1.7	0.48%	20.25%	210
*V. vulnificus*	High	≥2.7	9.38%	45.75%	11
	Medium	1.8–2.7	2.40%	29.85%	42
	Low	<1.8	0.28%	20.41%	358
2020–2024 (FDOH**)**					
Total vibriosis	High	≥2.5	46.99%	71.97%	2
	Medium	1.6–2.5	18.39%	22.69%	5
	Low	<1.6	3.27%	5.34%	31
*V. vulnificus*	High	≥2.7	16.43%	62.69%	6
	Medium	1.8–2.7	5.93%	23.88%	17
	Low	<1.8	1.14%	13.43%	88

**Fig 3:**
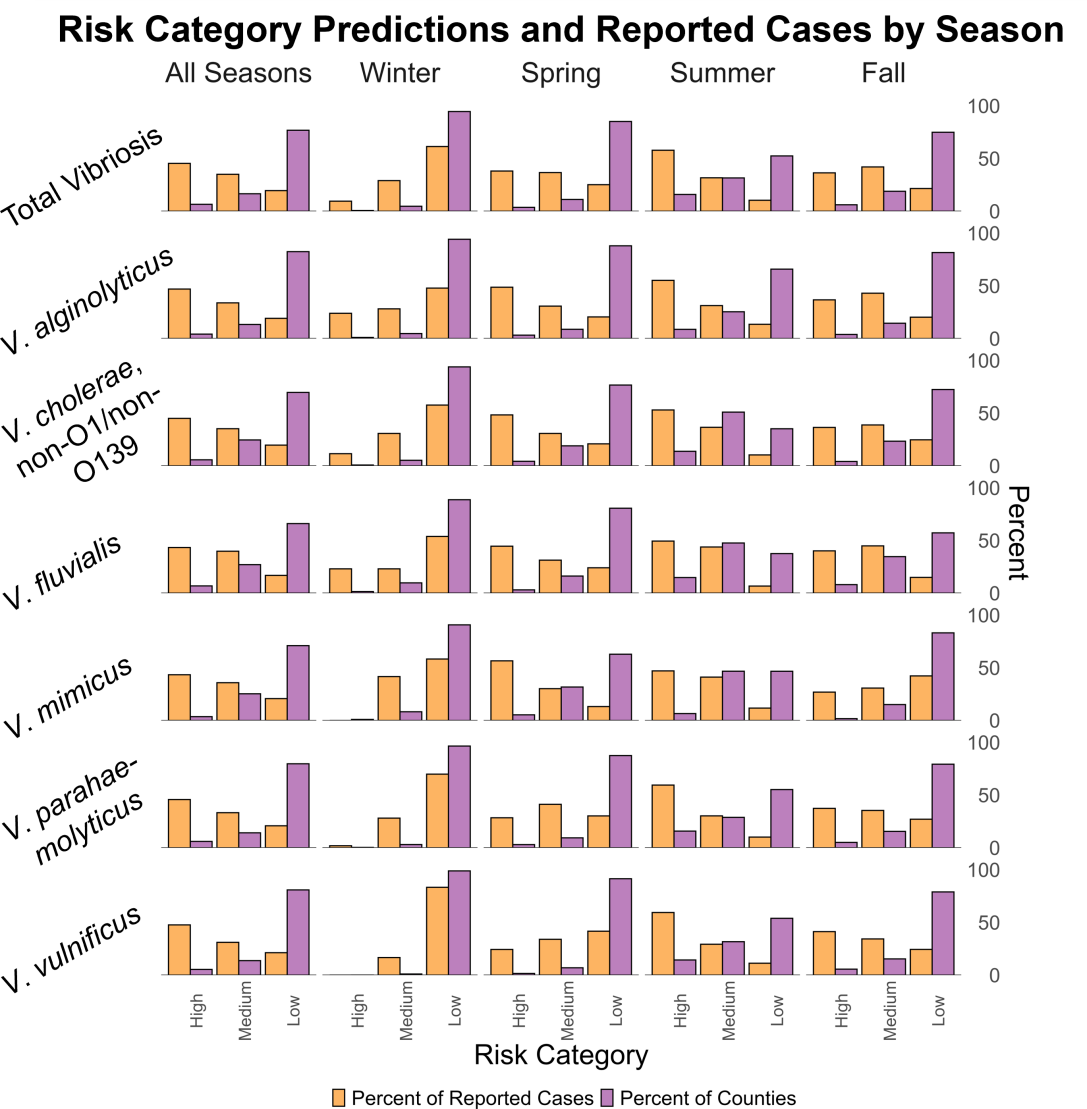
Distribution of reported cases and total predictions by predicted risk category, *Vibrio* spp., and season. Bar plots include percentage of reported cases (orange) and percentage of total predictions (purple) in each risk category for the season and species indicated. A total of 1,702 cases were reported for vibriosis in summer, 854 for fall, 679 for spring, and 241 for winter.

Models should identify a high percentage of cases in a low percentage of total counties for high risk, a low percentage of cases and a high percentage of counties for low risk, and a moderate percentage of both for medium risk. This trend is noted for all seasons, where high-risk predictions accounted for 43.4–47.7% of all reported cases in 3.63–6.89% of all counties, while low-risk predictions accounted for 16.9–21.3% of reported cases in 66.0–82.5% of all counties. The models identified more high-risk counties during the summer (June–August) and a greater proportion of cases in high-risk counties. There is a slight decrease in high-risk predictions during the spring (March–May) and fall (September–November) and a sharper decrease in winter (December–February), when most models rarely predicted high risk, likely because of infrequent case reports outside of summer. Figure 3 depicts the distribution of county-months identified for each risk category, as well as the number of cases for each risk category designation for each season.

### Validation results

**Fig 4:**
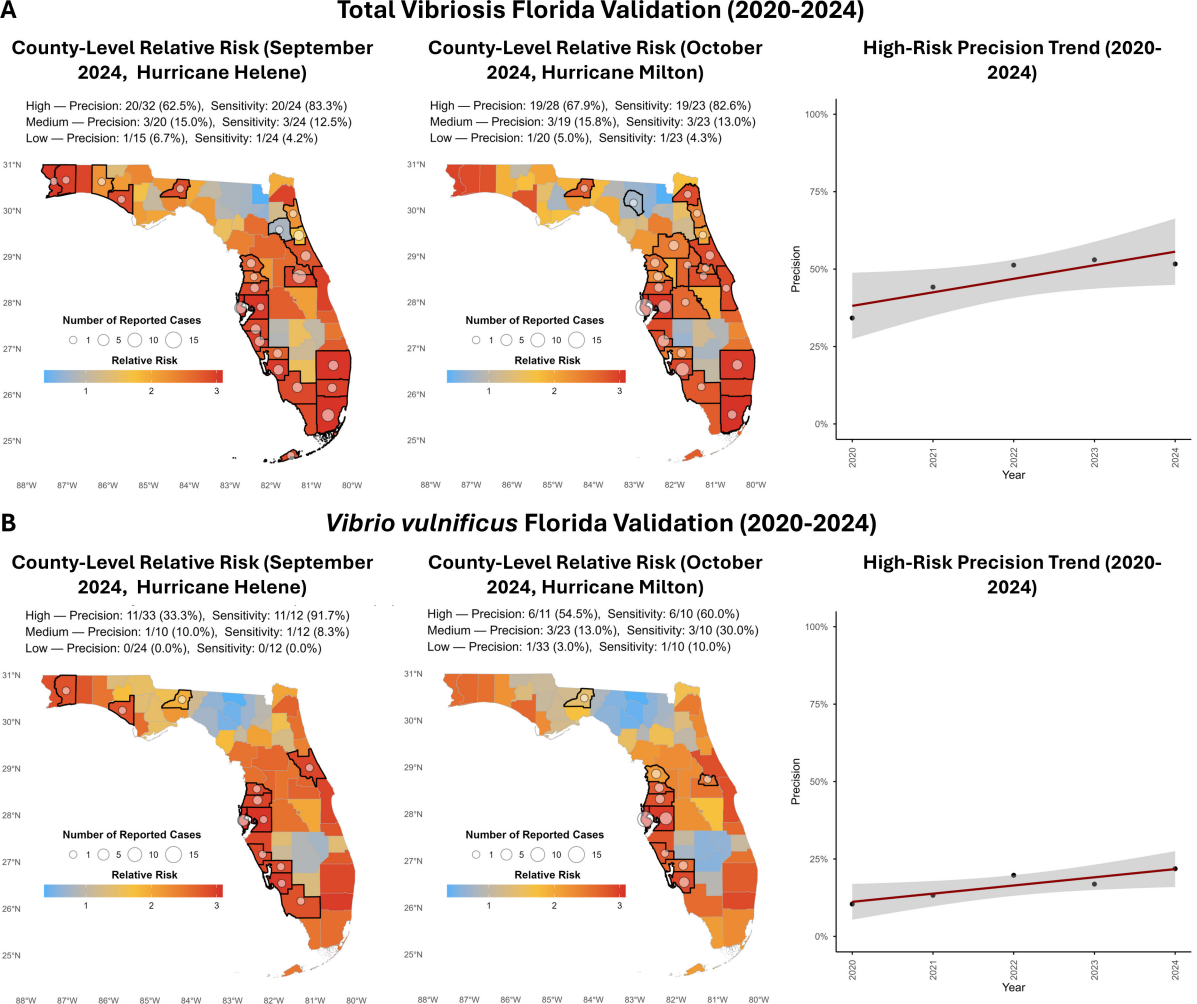
Validation results for (**A**) total vibriosis model and (**B**) *V. vulnificus* model. The maps display the number of reported cases and relative risk for (September and October 2024) (months of Hurricane Helene and Hurricane Milton), as well as the precision and sensitivity of risk category predictions by the model for those months. Trendlines (computed using linear regression) and 95% confidence interval (CI) depict annual precision of high-risk predictions for each model. The base maps are from the U.S. Census Bureau’s Cartographic Boundary Files.

Table 2 displays precision, sensitivity, and prediction-to-case ratio for Florida data validation of total vibriosis and *V. vulnificus* models. Interestingly, these two models performed better in validating 2020–2024 Florida vibriosis data than the original model using COVIS data. However, it should be noted that vibriosis models also performed better from 2007 to 2019 with the FDOH data than with COVIS data in Florida (see supplemental material for details). Nevertheless, the metrics for the FDOH data post-2019 exceeded pre-2019 performance for both data sets. Total vibriosis and *V. vulnificus* model annual precision improved significantly for high-risk prediction during 2020–2024, with *R*^2^ of 0.77 and 0.81, respectively (Fig. 4), though there is nonlinear interannual variation. Sensitivity for the 2020–2024 period also improved compared to the pre-2019 metrics.

In Florida, hurricanes are a serious concern. Outbreaks of disease caused by *Vibrio* spp. following Hurricane Katrina (86), Hurricane Ian (87), and more recently Helene and Milton in the Gulf Coast region of the United States are well documented (88). Therefore, special attention was given to predicted risk and reported cases of vibriosis during September and October of 2024, months when hurricanes Helene and Milton made landfall in Florida. Figure 4 displays risk model predictions and performance for those two months, with counties reporting cases. Results show that during hurricane months, 39 of 47 (83.0%) total vibriosis cases and 17 of 22 (77.2%) *V*. *vulnificus* cases were reported in counties identified as high risk. Conversely, only 2 of 47 (4.26%) total vibriosis cases and 1 of 22 (4.55%) *V*. *vulnificus* cases were reported in counties identified as low risk. As for precision, 39 of 60 (65.0%) of high-risk predictions by the total vibriosis and 17 of 44 (38.6%) predictions by the *V. vulnificus* models correctly corresponded to reported cases. These metrics for hurricane months exceed both the baseline metrics for pre-2019 testing and post-2019 validation. Also notable was the change in overall distribution of predictions, with distribution trending higher in September, when more cases were reported, and lower in October, with fewer cases reported. Additionally, counties predicted to be at higher relative risk, e.g., Pinellas and Hillsborough counties, reported the most cases during those two months.

### Precision trends

**Fig 5:**
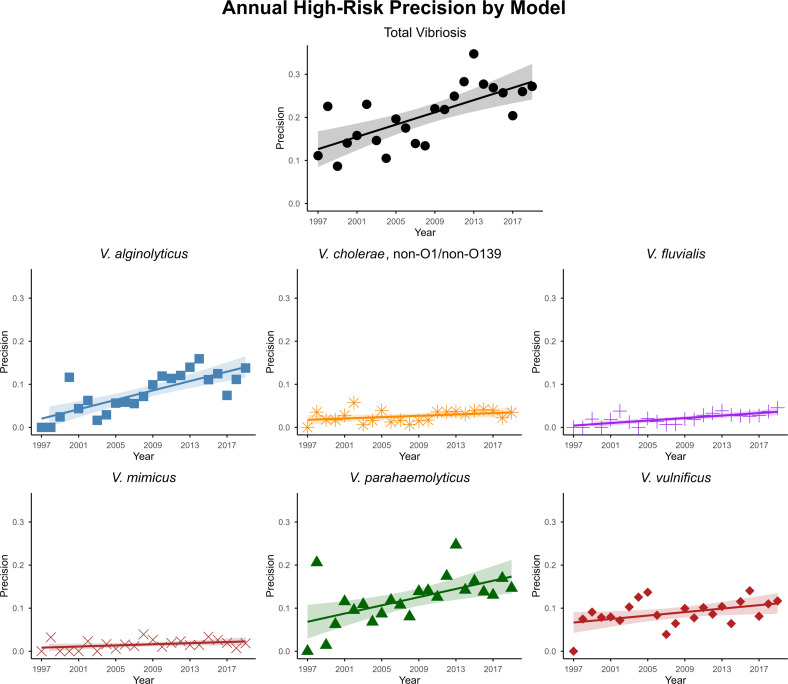
Annual precision of high-risk predictions for each model, including linear trendlines (computed using linear regression) and 95% confidence intervals.

Observation of improved precision in the validation study led to an analysis of annual trends of precision for all models, as shown in Fig. 5, including linear trendlines (computed with linear regression) and 95% confidence intervals showing increased precision for high risk over time, though most trends are nonlinear with significant interannual variation. Using the derived trendline equations, precision improved by 1.7–8.9 relative to baseline (equation intercept) by the end of the 23-year study period, depending on the model, with *V. cholerae* and *V. mimicus* improvement marginal and *V. alginolyticus* and *V. fluvialis* strong.

### Vibriosis risk and SSP scenario

**Fig 6:**
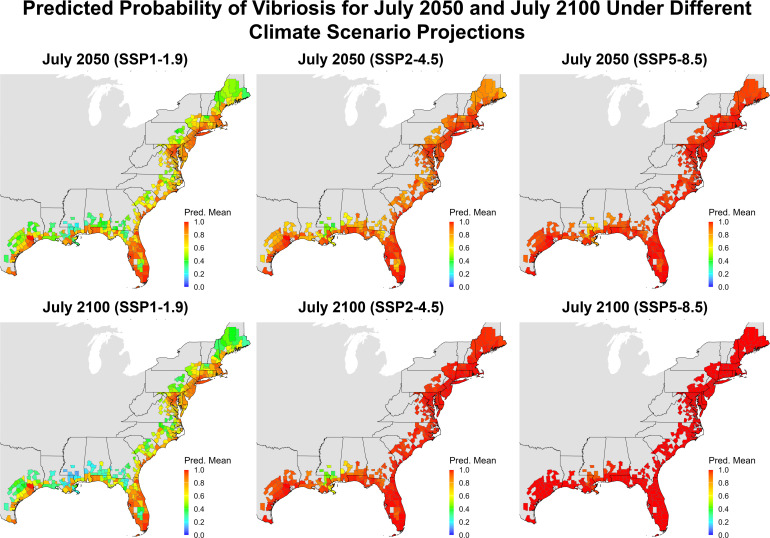
Projected predicted probability for total vibriosis using projected environmental data (fit with Bio-ORACLE v3.0 trends) under different Shared Socioeconomic Pathways (SSPs) for July 2050 and 2100. SSP1-1.9 is estimated to be 1.6°C warming by mid-century and 1.4°C by the end of the century, SSP2-4.5 is estimated to be 2.0°C warming by mid-century and 2.7°C by the end of the century, and SSP5-8.5 is estimated to be 2.4°C warming by mid-century and 4.4°C by the end of the century. Socioeconomic data were assumed constant (2019 baseline). Predicted probability is from 0 to 1 (0% to 100% probability of presence). The maps were created using the R package tigris.

Figure 6 displays ensemble-predicted probability of total vibriosis for July 2050 and 2100, using constant socioeconomic parameters. Sensitivity analysis (see supplemental material) demonstrated negligible difference using projected versus constant socioeconomic parameters for 2050 and 2100. Therefore, constant socioeconomic parameters were utilized due to high uncertainty and limiting assumptions from projecting socioeconomic parameters. All counties had ca. 100% predicted probability of vibriosis by the end of the century under SSP5-8.5, the worst-case climate scenario (2.4°C warming by mid-century and 4.4°C by the end of the century) (89). SSP2-4.5, a realistic projection for current climate action trend (2.0°C warming by mid-century and 2.7°C by the end of the century) (89), projected ca. 100% predicted probability for most counties by July 2050 and nearly all counties by July 2100. However, SSP1-1.9, considered the best-case climate action scenario (1.6°C warming by mid-century and 1.4°C by end of the century) (89), showed many counties at relatively low risk of vibriosis by July 2050 and even lower risk by July 2100. Other SSPs fell between the projections, with detailed maps and results provided in the supplemental material, with projected change in environmental parameters under each SSP scenario and baseline.

## DISCUSSION

The modeling results presented here demonstrate an effective strategy for forecasting vibriosis risk. Most models reported here utilized a combination of SST, DO, pH, and NO_3_⁻ as environmental indicators for vibriosis. Vibriosis cases showing positive association with SST and negative association with DO and NO_3_⁻ are in agreement with previous findings (2, 18, 25, 63, 64, 66). However, significant negative association with pH for most models merits comment. A review of environmental factors influencing *Vibrio* spp. in coastal environments found pH to be both positively and negatively associated with the incidence of *Vibrio* spp., depending on geographical area and *Vibrio* spp. (66). Previous modeling of *V. vulnificus* in the United States concluded pH not significant (47), as concluded by the *V. vulnificus* model (marginal significance of pH), whereas other models indicated pH to be a significant negative predictor for *Vibrio* spp. other than *V. vulnificus*, i.e., strong negative coefficient. It should be noted that alkaline pH is employed in laboratory culture to enhance growth and proliferation of *Vibrio* spp., notably *V. cholerae*. Hence, the pH range of an aquatic ecosystem can be growth-supportive rather than selective, i.e., enhanced growth and proliferation.

Socioeconomic variables, namely swim season days and total seafood establishments, contributed to a strong positive coefficient, indicating that modes of foodborne and nonfoodborne infection can be estimated by employing these parameters. It is interesting to note that *Vibrio* spp. associated with a higher rate of foodborne infection, compared to nonfoodborne infection, showed stronger contribution from seafood establishment totals rather than swim season days, and the inverse was also observed. Thus, the two parameters provide a potential estimate of the infection pathway. Also notable was population density having little impact on model prediction, with population clusters likely collinear with swim season days or total seafood establishments. Five models incorporated SVI, of which all had negative mean coefficients, with three significant. This finding is noteworthy as vulnerable populations may be underrepresented in vibriosis reporting due to lack of access to healthcare, hospitals, medical facilities, knowledge of infection, beaches, or seafood establishments. However, determining the precise cause will require further study.

Best-performing models, with respect to precision, were those for total vibriosis, *V. alginolyticus*, *V. parahaemolyticus*, and *V. vulnificus*. All models showed increased precision over time, with varying magnitude of improvement. The trends went beyond the 1997–2019 calibration period, with increased precision for high-risk predictions during 2020–2024 for Florida total vibriosis and *V. vulnificus*. Enhanced monitoring and reporting of *Vibrio* spp. cases likely contributed to performance gain. The models also showed strong promise with respect to extreme events, namely hurricanes. During September and October 2024, when hurricanes Helene and Milton swept across Florida, the precision of high-risk prediction was much higher than the precision of both calibration and total validation period. Additionally, relative risk was highest for Tampa Bay (Pinellas and Hillsborough counties) during hurricane months, with most cases reported as occurring during the events. As public health surveillance of vibriosis improves and remote sensing technologies for environmental monitoring continue to advance, the models presented here will be more precise and valuable for informed decision-making.

Risk of vibriosis, based on model prediction, increased, with predicted annual probability of cases nearly doubling between 1997 to 2019 for all *Vibrio* spp. included in the study. The models captured changes in baseline risk without employing an annual temporal variable, with variables contributing to increased vibriosis risk over time, reflecting year-to-year changes in environmental and socioeconomic conditions. This conclusion supports the previously reported findings of increased cases in the eastern United States aligned with environmental change (12, 13, 21, 24, 25). Many reports also aligned with the hotspots identified here, including areas of high average vibriosis risk, namely Florida and the Gulf Coast, as well as Long Island, New York, eastern Massachusetts, southwest Connecticut, both Savannah, Georgia, and Charleston, South Carolina, and the northern Chesapeake Bay.

Nonlinear interannual variation is noted in both model precision and predicted probability, and these trends may be partially driven by the El Niño–Southern Oscillation (ENSO) and Atlantic Meridional Overturning Circulation (AMOC), as long-term climate oscillations have been associated with presence of cholera (90). Using data obtained from the NOAA Climate Prediction Center (91) and Met Office (92), annual correlations of ENSO and AMOC with total vibriosis presence, model precision, and predicted probability were computed. ENSO appears to have minimal impact on this noted interannual variation, with weak and insignificant positive correlation with presence (*r* = 0.211, *P* = 0.132), precision (*r* = 0.306, *P* = 0.078), and predicted probability (*r* = 0.273, *P* = 0.104). AMOC had insignificant negative correlation with presence (*r* = −0.359, *P* = 0.086) and predicted probability (*r* = −0.213, *P* = 0.214), but significant negative correlation with model precision (*r* = −0.464, *P* = 0.035). AMOC data availability limits the temporal range of the analysis (2004–2019), so it is difficult to determine the extent of this association, but it is possible that increases in vibriosis case reports may be linked to these multi-decadal climate patterns. The model appears to capture the local vibriosis report impacts of these globalized climate shifts despite their absence in the models, indicating the strength of using the INLA-SPDE framework.

Although nonlinear, the general trend of increasing predicted probability is predicted to continue into the future unless major climate action is taken to reduce greenhouse gas emissions, as demonstrated by model results under varying climate scenarios presented here. SSP5-8.5, the worst-case scenario of extreme emissions and no policy change, presents a bleak future for vibriosis risk by 2050 and 2100. However, it is unlikely and only demonstrates the worst-case impact of no climate action (93). Unfortunately, SSP1-1.9, which meets the Paris Climate Agreement goals and shows lower risk of vibriosis for 2050 and 2100, is unlikely to occur given the current trajectory of policies (93). SSP2-4.5 is considered the most realistic best-case outcome of moderate mitigation (though surpassing 2.5°C warming compared to 1850–1900) (89, 93), yet even it presents most coastal counties at ca. 100% predicted probability of vibriosis by July 2050 and nearly all coastal counties ca. 100% predicted probability by July 2100. The SSP4-6.0 scenario (weak mitigation policies), similarly likely based on current policies (93), shows similar results to SSP2-4.5. Overall, increased risk of vibriosis can be expected for the remainder of the 21st century without a change in climate mitigation policy.

### Study limitations

The primary limiting factor of this study was the uncertainty of location of both case initiation and the proximity of cases to given environmental conditions. That is, cases were assumed to have been accurately reported in the county of interest, but with possible lagged reporting of cases, unreported cases, or cases having no association with source environmental conditions, such as imported seafood or hospital visits not in the county where the case occurred. This issue became more apparent when examining discrepancies between national-level reporting (COVIS) and local reporting (FDOH). These factors limit precision; hence, excluding patient travel from the case data reduced uncertainty. However, these limitations do not appear to have impacted model training, as the models show improved performance with FDOH data compared to COVIS data for 2007–2019, despite not having been trained on FDOH data. This indicates that national reporting is sufficient for the models to detect environmental and socioeconomic signals of vibriosis cases, even if national reports are noisy or not precise. Also, previous studies showed minimal differences for foodborne and nonfoodborne case modeling of COVIS using environmental variables (25), so inclusion of both did not impact results significantly.

COVIS reporting methods changed since 2017 to include cases identified using culture-independent diagnostic tests (CIDTs) (94). Not controlling for this change in reporting from 2017 to 2019 may be responsible for the increased predicted probability and precision of the models, since it is a generally more accurate method. However, trends in probability and precision are noted prior to 2017; thus, the impact of the change on the results is difficult to quantify.

Socioeconomic data were not available for all years of the calibration period; therefore, linear trends were employed. Ideally, data for all years would be available. However, model performance would likely show very small improvement with such data, since the precision improvement with additional variables was small. Similarly, more accurate future predictions of socioeconomic variables are unlikely to alter the main findings of the 2050 and 2100 projections, as the sensitivity analysis demonstrated minimal change between results for both constant (2019) and projected variables utilizing linear trends.

Using 1-month lagged environmental variables also presents a limitation, as not all variables may have maximum predictive power with a 1-month lag. However, computational requirements for testing all variables at several different lagged combinations on such a large data set made 1-month lag an appropriate decision. Improvements in the future with respect to the combination of lag times will likely be marginal.

The models were calibrated and validated for eastern coastal areas of the United States. While the same parameter data sets may be used to forecast vibriosis risk in the western coastal United States, validation of the models would still be necessary. Application of the models globally will require socioeconomic data, swim season days, total seafood establishments, population density, and SVI. The challenge will be to have standardized variables. Methodology to calculate each is available (from data source) in order to validate models for other areas of the world. Alternatively, the methodology outlined here can be used to build predictive models that can be calibrated for application.

### Conclusions

With increasing reports of vibriosis in the eastern United States, there is a need for predictive intelligence for these illnesses. Here, vibriosis prediction models for *V. alginolyticus*, *V. cholerae* non-O1/non-O139, *V. fluvialis*, *V. mimicus*, *V. parahaemolyticus*, *V. vulnificus*, and the combined species provide insight for vibriosis mitigation and intervention. Notably, models for *V. alginolyticus*, *V. parahaemolyticus*, *V. vulnificus*, and combined vibriosis provide precision of >9%, performing best during peak vibriosis season, notably summer months, and improving over time. This allows prediction of vibriosis up to 1 month in advance for high-risk U.S. counties. Notably, analyses of data for Florida show that the models can be utilized effectively during extreme weather events, namely hurricanes. Predictions derived from the models indicate increasing probability of vibriosis over time, with the probability of vibriosis nearly doubling between 1997 and 2019, and projected to reach ca. 100% during peak season for most counties in the eastern United States by 2100 under currently likely climate scenarios. In summary, based on the results of the study reported here and significant changes occurring in environmental conditions along coastal areas of the eastern United States, the development of an early warning system to assist in mitigating the rising risk of vibriosis is warranted.

## AFTER EPUB

*[This article was published on 11 March 2026 with the incorrect graphic for Fig. 6. The figure was updated in the current version, posted on 12 March 2026.]*

## SUPPLEMENTAL MATERIAL

Supplemental material aem.02135-25-s0001.docx Fig. S1 to S4; Tables S1 to S3.

ASM does not own the copyrights to Supplemental Material that may be linked to, or accessed through, an article. The authors have granted ASM a non-exclusive, world-wide license to publish the Supplemental Material files. Please contact the corresponding author directly for reuse.
