# Supplementary material for: Predictive intelligence for future vibriosis risk in the eastern United States employing Bayesian spatial modeling
Source: Appl Environ Microbiol. 2026 Mar 11;92(4):e02135-25. doi: 10.1128/aem.02135-25 (PMC13101486; doi:10.1128/aem.02135-25)
Supplement: Supplemental material — Fig. S1 to S4; Tables S1 to S3. [file aem.02135-25-s0001.docx]

**Table S1.** Demographic data for each environmental and socioeconomic parameter included in the models. Environmental data points per month include the number of satellite/modeled observations included in the 20 km area of the eastern and southern coastline of the U.S.

| Parameter | Number of Environmental Data Points Per Month | Mean | SD | Min | Q1 | Median | Q3 | Max |
| --- | --- | --- | --- | --- | --- | --- | --- | --- |
| CDOM (mmol/m^3^) | 5731 | 4.52E-01 | 3.64E-01 | 9.00E-03 | 1.71E-01 | 3.65E-01 | 6.33E-01 | 4.29E+00 |
| DO (mmol/m^3^) | 260 | 235 | 14.4 | 205 | 224 | 233 | 244 | 287 |
| Micro-phytoplankton (mg/m^3^) | 8376 | 9.86 | 6.13 | 0.01 | 5.09 | 8.97 | 13.6 | 40.5 |
| Nano-phytoplankton (mg/m^3^) | 8376 | 0.218 | 0.203 | 0.000 | 0.000 | 0.187 | 0.415 | 0.601 |
| NO3 (mmol/m^3^) | 260 | 1.61 | 2.49 | 0.00 | 0.04 | 0.48 | 2.01 | 14.5 |
| pH | 260 | 8.07 | 0.03 | 7.99 | 8.05 | 8.07 | 8.09 | 8.14 |
| Pico-phytoplankton (mg/m^3^) | 260 | 2.17E-01 | 1.93E-01 | 1.60E-02 | 8.80E-02 | 1.45E-01 | 2.70E-01 | 1.09E+00 |
| PO4 (mmol/m^3^) | 8376 | 4.60E-02 | 9.20E-02 | 0.00E+00 | 0.00E+00 | 0.00E+00 | 2.90E-02 | 3.29E-01 |
| Precipitation (m^3^) | 273 | 3.00E-03 | 2.00E-03 | 0.00E+00 | 2.00E-03 | 3.00E-03 | 4.00E-03 | 2.50E-02 |
| SSS (ppt) | 252 | 29.5 | 7.58 | 7.81 | 27.8 | 32.2 | 34.9 | 36.6 |
| SST (˚C) | 230 (AVHRR)/ 8367 (MODIS) | 19.2 | 8.4 | -1.7 | 13.1 | 20.0 | 26.5 | 34.7 |
| Population density (Population/mi^2^) | - | 1046 | 4542 | 4.26 | 55.9 | 157 | 487 | 74288 |
| Social Vulnerability Index | - | 0.57 | 0.29 | 0.00 | 0.32 | 0.62 | 0.83 | 1.00 |
| Swim season days | - | 61.3 | 106 | 0.00 | 0.00 | 0.00 | 98.0 | 365 |
| Seafood establishments | - | 6.53 | 14.4 | 0.00 | 0.00 | 2.00 | 7.00 | 180 |


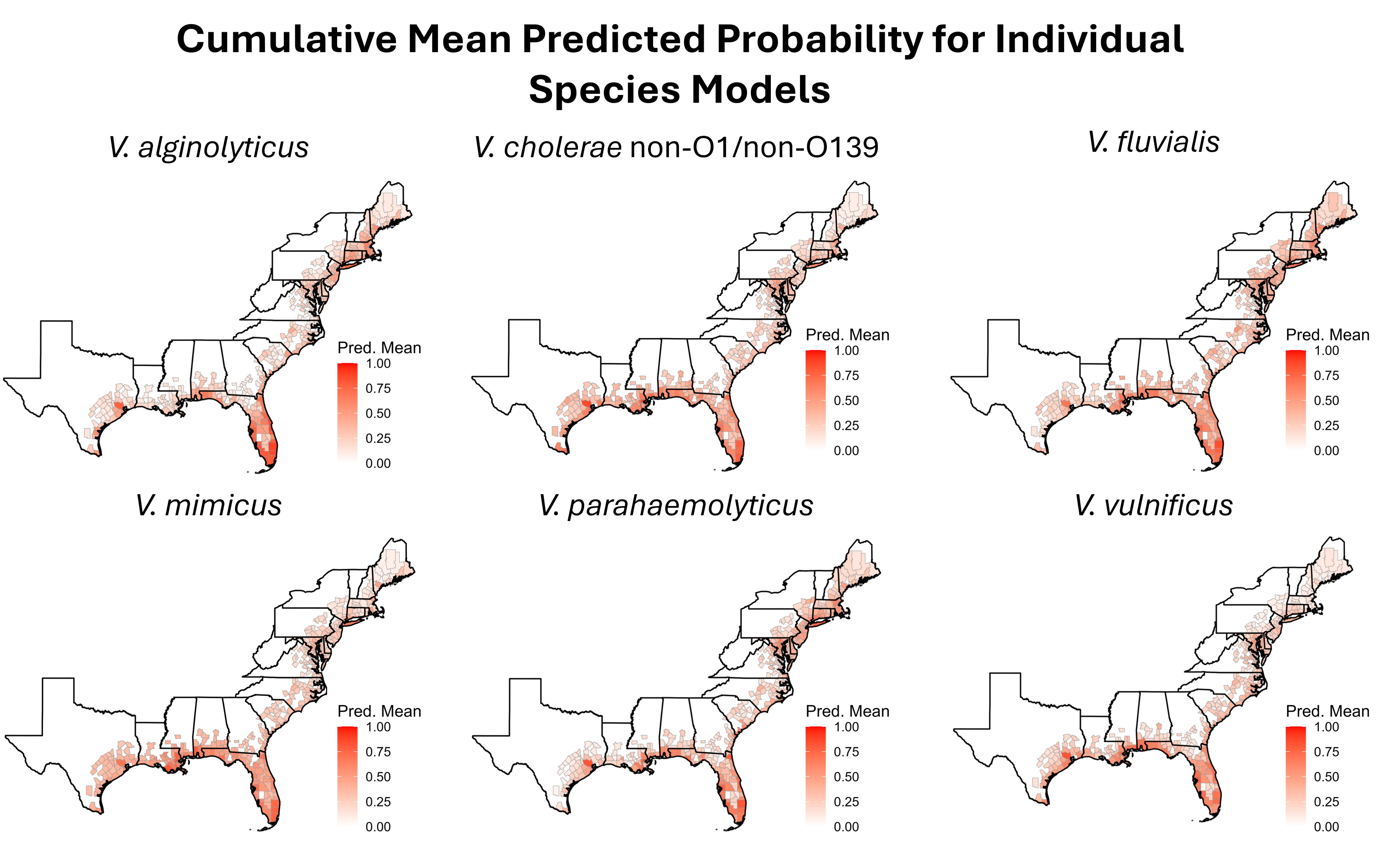


**Figure S1.** Cumulative mean predicted probability of vibriosis for individual *Vibrio* spp. models, with predicted probability from 0 to 1 (0 % to 100 % probability of presence). Includes all testing data predictions for all available counties (n = 405 counties).


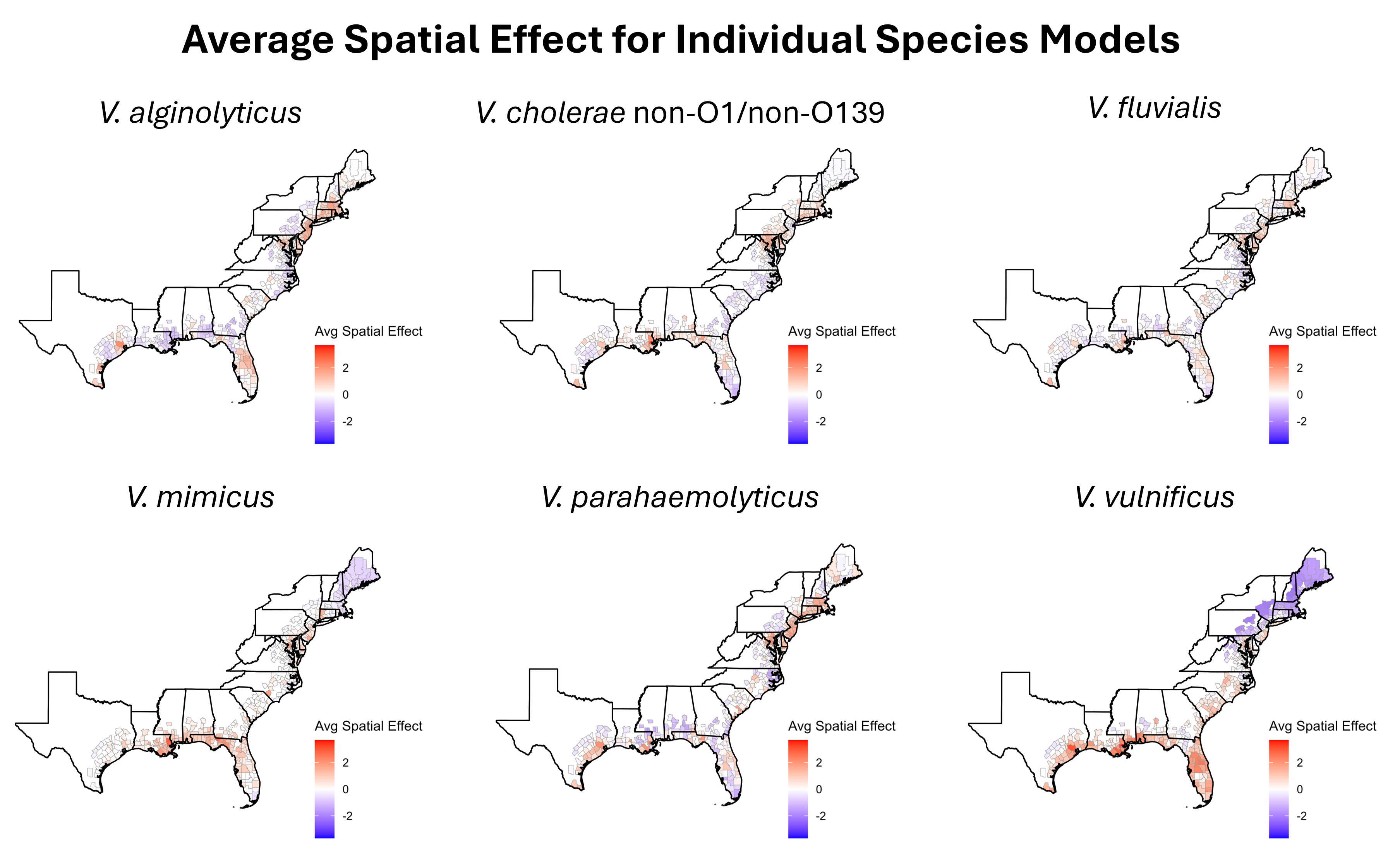


**Figure S2.** Cumulative mean spatial random effects for individual *Vibrio* spp. models. Spatial effects represent residual spatial autocorrelation not explained by measured environmental variables. Values indicate areas with higher or lower vibriosis risk than expected based on fixed predictors.

**Table S2.** Comparison of model performance and risk category distribution for COVIS and FDOH vibriosis case reports for total vibriosis and *V. vulnificus* (2007-2019). Presence is defined as a case reported within a county-month, while absence is defined as no case being reported within a county-month.

|  | **Total Vibriosis** | | | ***V. vulnificus*** | | |
| --- | --- | --- | --- | --- | --- | --- |
| Modeled Risk Category | High | Low | Medium | High | Low | Medium |
| Number of County-Months | 1922 | 5170 | 2672 | 1613 | 6087 | 2064 |
| Number of Same Presence/ Absence Observations | 1396 | 4804 | 2286 | 1463 | 5913 | 1948 |
| FDOH Case Presence Only | 407 | 134 | 263 | 115 | 44 | 65 |
| COVIS Case Presence Only | 119 | 232 | 123 | 35 | 130 | 51 |
| Percent Same Presence | 42.6% | 26.7% | 33.7% | 34.5% | 14.7% | 34.8% |
| Percent Same Absence | 65.7% | 92.7% | 84.4% | 90.2% | 97.1% | 94.2% |
| Percent Same Total | 72.6% | 92.9% | 85.6% | 90.7% | 97.1% | 94.4% |
| COVIS Precision | 26.5% | 11.9% | 7.06% | 7.07% | 5.47% | 2.63% |
| COVIS Sensitivity | 42.7% | 26.7% | 30.6% | 29.5% | 29.2% | 41.3% |
| FDOH Precision | 41.5% | 17.2% | 5.16% | 12.0% | 6.15% | 1.22% |
| FDOH Sensitivity | 52.3% | 30.1% | 17.5% | 49.1% | 32.2% | 18.7% |

**Table S3.** Summary statistics for absolute value residuals in predicted probability values for all counties using constant (2019 baseline) versus projected (with linear trends) socioeconomic variables. These statistics include predictions for all six SSPs employed for scenario generation.

|  | Mean | SD | Min | 25th | Median | 75th | Max |
| --- | --- | --- | --- | --- | --- | --- | --- |
| Value | 7.29E-03 | 2.01E-02 | 0.00 | 0.00 | 5.85E-04 | 5.43E-03 | 3.10E-01 |


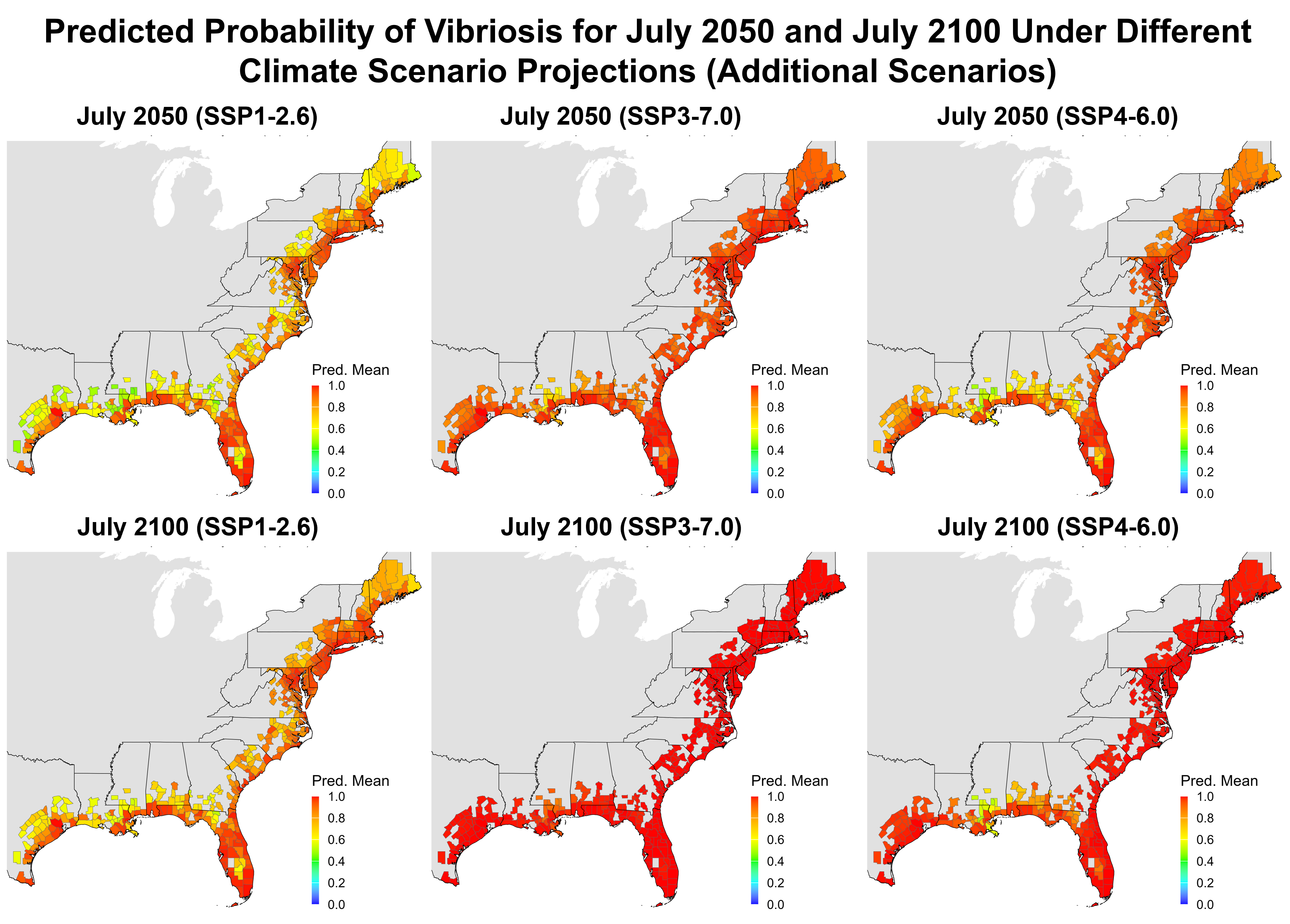


**Figure S3.** Projected predicted probability for total vibriosis using projected environmental data (fit with Bio-Oracle V3.0 trends) under different Shared Socioeconomic Pathways (SSPs) for July 2050 and 2100. SSP1-2.6 is estimated to be 1.7 ˚C warming by mid-century and 1.8 ˚C by the end of the century, SSP3-7.0 is estimated to be 2.1 ˚C warming by mid-century and 3.6 ˚C by the end of the century, and SSP4-6.0 is estimated to between SSP2-4.5 and SSP3-7.0 (warming estimate not given in IPCC AR6 for this SSP because it is considered a tier 2 scenario). Socioeconomic data were assumed constant (2019 baseline). Predicted probability is from 0 to 1 (0 % to 100 % probability of presence).


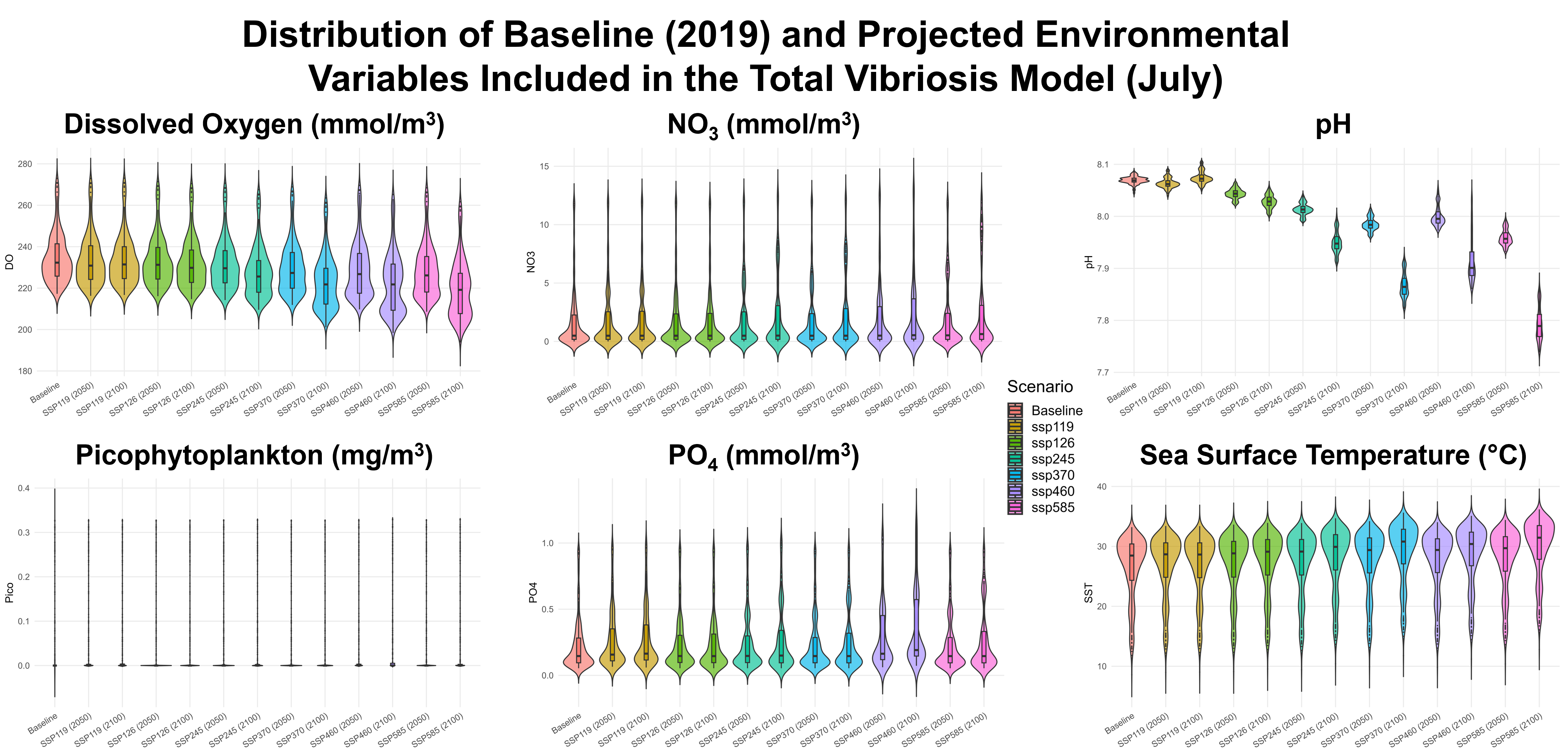


**Figure S4.** Violin plots of environmental variables included in the total vibriosis model, including baseline distribution (July 2019) and future projections under different SSP scenarios. Variable projections were computed using county-level linear trends from Bio-Oracle v3.0 data with linear trends applied to the original calibrated datasets. The violin plots depict box and whisker plots of each SSP and associated year’s distribution, and the shaded width area indicates the relative volume of data points along each part of the distribution to visualize data point concentration.
